# Impact of experimental setup parameters on the measurement of articular cartilage optical properties in the visible and short near-infrared spectral bands

**DOI:** 10.1364/BOE.488801

**Published:** 2023-06-15

**Authors:** Iman Kafian-Attari, Ervin Nippolainen, Florian Bergmann, Arash Mirhashemi, Petri Paakkari, Florian Foschum, Alwin Kienle, Juha Töyräs, Isaac O. Afara

**Affiliations:** 1 Department of Technical Physics, University of Eastern Finland, Finland; 2 Diagnostic Imaging Center, Kuopio University Hospital, Finland; 3 Institute for Laser Technologies in Medicine and Meteorology, University of Ulm, Germany; 4 Science Service Center, Kuopio University Hospital, Finland; 5 School of Information Technology and Electrical Engineering, University of Queensland, Australia

## Abstract

There is increasing research on the potential application of diffuse optical spectroscopy and hyperspectral imaging for characterizing the health of the connective tissues, such as articular cartilage, during joint surgery. These optical techniques facilitate the rapid and objective diagnostic assessment of the tissue, thus providing unprecedented information toward optimal treatment strategy. Adaption of optical techniques for diagnostic assessment of musculoskeletal disorders, including osteoarthritis, requires precise determination of the optical properties of connective tissues such as articular cartilage. As every indirect method of tissue optical properties estimation consists of a measurement step followed by a computational analysis step, there are parameters associated with these steps that could influence the estimated values of the optical properties. In this study, we report the absorption and reduced scattering coefficients of articular cartilage in the spectral band of 400-1400 nm. We assess the impact of the experimental setup parameters, including surrounding medium, sample volume, and scattering anisotropy factor on the reported optical properties. Our results suggest that the absorption coefficient of articular cartilage is sensitive to the variation in the surrounding medium, whereas its reduced scattering coefficient is invariant to the experimental setup parameters.

## 1. Introduction

The healthy function of the human musculoskeletal system depends on a plethora of biomechanical cues, including the ability of tissues, such as articular cartilage, to dissipate mechanical loading to the underlying bone and regular maintenance of their extracellular matrix by their resident cells [1]. Among the connective tissues of the articulating joints, articular cartilage is the most affected by osteoarthritis. In the case of posttraumatic osteoarthritis [2], osteoarthritic articular cartilage exhibits cell inflammation, extracellular matrix disintegration, disrupted osmolarity, initial swelling of the matrix, and gradual loss of matrix and mechanical strength [3]. Articular cartilage comprises water, collagen and elastin fibers, proteoglycans, chondrocytes (cell population), and minor mineral compounds. Osteoarthritis disrupts the homeostatic relationship between collagen fibers and proteoglycans – two macromolecules that determine and regulate the mechanical properties of articular cartilage [4]. Collagen fibers form the structural framework of the articular cartilage extracellular matrix and assume a logical layered structure due to their depth-varying orientation. In the superficial region of the tissue, collagen fibers have parallel alignment to the tissue surface, and their orientation gradually skews to perpendicular alignment to the tissue surface in the deeper regions of the tissue matrix [1].

Diagnosis of articular cartilage injuries, their severity, and their extent is critical for effective repair operation and prevention of posttraumatic osteoarthritis [5]. To this end, there has been growing interest in the research domain to develop objective methods for rapid, quantitative, and intraoperative evaluation of articular cartilage health to distinguish osteoarthritic tissue from healthy tissue. Biomedical optical methods, including diffuse optical spectroscopy, hyperspectral imaging, fluorescence spectroscopy, and Raman spectroscopy, have shown the potential to become ideal candidates for such applications [6–12].

In diffuse optical spectroscopy, the interaction of light in biological tissues, and subsequently the optical response of tissues, is governed by intrinsic fundamental and wavelength-dependent properties (optical properties), including absorption coefficient ( $\mu_{a}$ ), single scattering coefficient ( $\mu_{s}$ ), scattering anisotropy factor ( g ), reduced scattering coefficient ( $\mu_{s}^{\prime }=\mu_{s}(1-g)$ ), and refractive index ( n ) of biological tissues [13]. Key factors determining the optical properties of biological tissues, especially absorption and scattering coefficients, are concentration, size, distribution, and alignment of the cells and macromolecules that form the solid tissue matrix. The optical properties of biological tissues, particularly the absorption coefficient in the near-infrared (NIR) spectral range, have been adopted as biomarkers for the non-invasive screening of tissue pathologies in multiple organs, including muscles, brain, skin, heart, and breast [13]. It is noteworthy to mention that another optical method, optical coherence tomography (OCT), which is based on the measurement of the backscattered intensity of NIR coherent light, has the potential for the detection of early-stage osteoarthritis. The intensity of the backscattered light is linked to tissue optical properties via the attenuation coefficient ( $\mu_{t}=\mu_{a}+\mu_{s}$ ). Studies [14–17] show that the OCT signal is correlated with histological and biomechanical properties of articular cartilage in all degrees of degeneration and health. Recent OCT studies focused on the surface irregularities such as fibrillation, cracks, and fissures as early signs of osteoarthritis. In addition, OCT combined with optical clearing enables estimation of articular cartilage subchondral bone [18] and the polarization-sensitive OCT (PS-OCT) has the potential to assess changes in cartilage collagen alignment by tracking the alteration of birefringence in articular cartilage.

In contrast, in musculoskeletal research, little attention has been paid to the ability of tissue optical properties to characterize changes in the biomolecular properties of articular cartilage during degeneration. Degenerative diseases like osteoarthritis affect the extracellular matrix of connective tissues, such as articular cartilage. The disease affects the quantity and orientation of the collagen fibers, as well as the tissue’s proteoglycan and water contents. These changes, which affect the physical properties of the tissue, will ultimately result in the alteration of the absorption coefficient and reduced scattering coefficient of the tissue. For instance, osteoarthritis results in increased matrix water content, which will concomitantly alter the absorption profile of the tissue. Similarly, disruption of the collagen network, the major solid component of the tissue, is likely to affect the scattering properties and ultimately result in the alteration of the tissue’s reduced scattering coefficient. As collagen fibers are photon scatterers, altering their concentration and orientation will change how light is scattered through the tissue. Thus, these disease-induced changes in matrix constituents will modify how much light is absorbed, reflected, and transmitted through the tissue. Subsequently, this will affect the reflectance and transmittance of light from the tissue, leading to changes in absorption and reduced scattering coefficients. In the past two decades, sporadic studies have tried to determine the optical properties of cartilage tissues from different anatomical locations of various animal species [19–26]. Given recent developments related to the precise determination of optical properties in Foschum et al. [27,28], the present study revisits the estimation of optical properties of articular cartilage from different anatomical locations of the bovine knee by utilizing the integrating sphere setup developed in Foschum et al. [27,28].

This study is composed of two parts, and it aims to provide accurate values of $\mu_{a}$ and $\mu_{s}^{\prime }$ of articular cartilage from different anatomical locations within the knee over the spectral band of 400-1400 nm. As every indirect method of tissue optical properties estimation consists of a measurement step followed by a computational analysis step, there are parameters associated with these steps that could influence the estimated values of the optical properties. In the first part of this study, we assess the impact of the surrounding medium filling the lateral gap between the sample and the inner walls of the sample holder, the physical volume of the samples, and the scattering anisotropy factor. These parameters are referred to as setup parameters throughout the text. To test the accuracy of the estimated optical properties of articular cartilage, we compare the results with the values reported in the literature. In the second part of this study, we use the Monte Carlo technique in combination with the optical properties in the present report. The objective is to estimate the depth of signal origin from different anatomical locations. Furthermore, we investigate the volume fraction of Mie- and Rayleigh scatterers and assess their impact on the scattering phase function and simulated reflectance and transmittance.

## 2. Methods

### 2.1 Sample preparation

Bovine knee joints ( n=15 ), collected from a local abattoir within one week of slaughter, were used in this study. No ethical permission was required. To prevent any biological deterioration, the joints were preserved in a vacuum bag and kept at 4 ∘C before the harvesting procedure (less than 48 hours). Osteochondral plugs were harvested from the lateral and medial sides of the articulating surfaces of the distal femur, proximal tibia, and patella using a stand-alone drilling system with a cylindrical drill bit (inner diameter 14 mm). During the process, the cartilage surface was continuously rinsed with phosphate-buffered saline (PBS, pH 7.4) to prevent interstitial water evaporation, osmolarity disruption, or change in the pH of the samples. A total of 68 samples were collected from lateral femoral condyle (FL, n = 14), medial femoral condyle (FM, n = 14), lateral patella (PL = 12), medial patella (PM, n = 11), lateral tibia (TL, n = 9), and medial tibia (TM, n = 8). Furthermore, the bone end of each sample was filed until it was parallel to the articular surface. The choice of bovine articular cartilage in this study was primarily due to ease of access and also due to difficulties in accessing healthy human cartilage. Nevertheless, given the compositional and structural similarities between human and bovine cartilage, we believe that the trends in the optical property will be similar.

### 2.2 MicroCT imaging

After extraction, the osteochondral samples were imaged with a microCT scanner (XTH 225, Nikon Metrology, Leuven, Belgium) in order to estimate the thickness ( mm ), surface diameter ( mm ), and volume ( $\text{m}{\text{m}}^{3}$ ). Images were acquired with $40\;\times \;40\;\times \;40\;\mu {\text{m}}^{3}$ isotropic voxel size, but the voxel size was increased to $50\;\times \;50\;\times \;50\;\mu {\text{m}}^{3}$ when reconstructions were calculated. The tube voltage was set to 80 kVp and the tube current to 375 μA with a 1.0 mm aluminum filter [29].

**Fig. 1.**
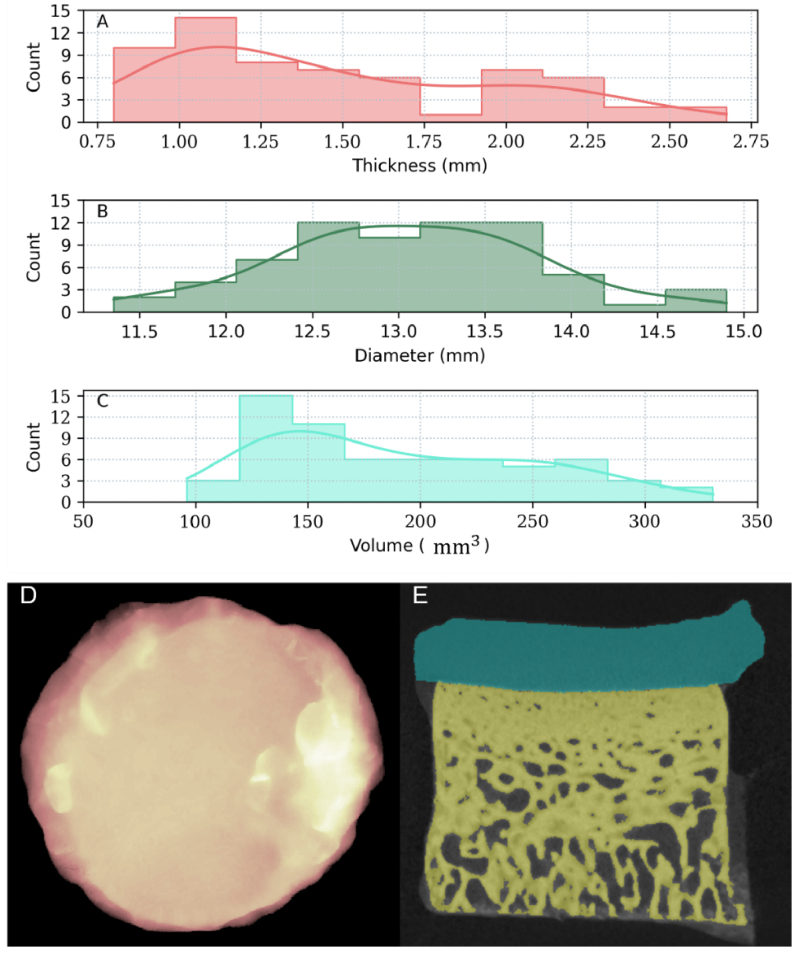
The distribution of the physical properties of the articular cartilage samples, estimated from the microCT images. (A) thickness ( mm ); (B) surface diameter ( mm ); (C) volume ( $\text{m}{\text{m}}^{3}$ ); (D) the top surface of an articular cartilage sample after segmentation; and (E) the X-Z cross-section of the sample which is segmented to articular cartilage (cyan color), bone (yellow color), and background medium (black color). The error of the articular cartilage volume segmentation from the microCT images was approximately less than 18%.

After image acquisition and reconstruction, the surface diameter and thickness of the cartilage segment of the osteochondral samples were estimated from the x-z and y-z planes that go through the midpoint of the samples. Furthermore, the 3D osteochondral microCT images were segmented to obtain the volume of the articular cartilage segment. Considering that the microCT image histogram has three peaks, it can be readily segmented into three classes using histogram thresholding. The classes are bone, soft tissue (articular cartilage and moisture), and background (including air and the sample holder). Distinguishing moisture from articular cartilage is not possible using their intensity values alone. Therefore, the segmentation was using morphological operators and surface normal vectors to geometrically isolate the articular cartilage tissue from the moisture. Figure 1 depicts the thickness, surface diameter, and volume of the osteochondral samples estimated by processing the microCT images. After careful examination of the segmentation and testing different scenarios, we consider the segmentation error not to exceed more than 18% of the sample volume.

After microCT imaging, the articular cartilage portion of the samples was mechanically detached from the subchondral bone by using a scalpel, and then the samples were stored in PBS at -20 ^°^ C .

### 2.3 Optical measurement and estimation of articular cartilage optical properties

**Fig. 2.**
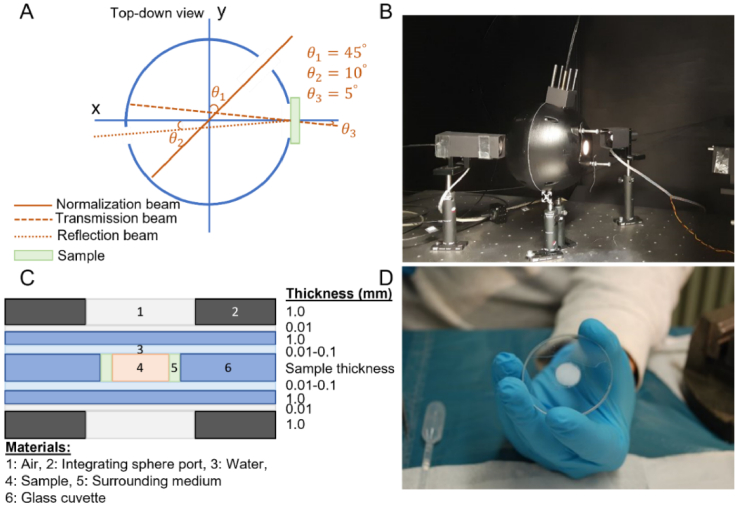
Schematics of the integrating sphere setup and sample holder used for optical measurement. (A) and (B) show the schematic and image of the integrating sphere setup, respectively. (C) shows the schematic of the sample holder and all the associated material properties and their physical dimensions. (D) shows an image of a cartilage sample embedded in the holder before optical measurement.

Before optical measurement, the samples were thawed at room temperature for 30 minutes. Subsequently, they were placed within a cylindrical sample holder of glass cuvettes to reduce the refractive index mismatch between the tissue and the surrounding medium (Fig. 2(C) and 2(D)). Afterward, an optimized double-beam single integrating sphere setup (Fig. 2(A) and 2(B)) was used to measure the reflectance and transmittance in the spectral band of 400-1400 nm. The integrating sphere setup was equipped with a halogen light source (Halostar Starlite, OSRAM, Germany), and two spectrometers, one for the visible spectral range with an approximate resolution of 3 nm (Maya2000Pro, Ocean Optics, USA) and one for the near-infrared band (NIRQuest512-1.7, Ocean Optics, USA) with an approximate resolution of 7 nm. Details of the optimized integrating sphere setup and refractive index matching sample holder are outlined in the literature [26–28]. Minimal absorption of light by water in the spectral band of 400-1400 nm, allows the spectral features of other chromophores present in biological tissues to be detectable. Moreover, although biological tissues exhibit relatively strong scattering properties in this spectral band when combined with the forward propagation nature of light in biological tissues, a high penetration depth can be achieved. Hence, the optical signals (reflectance and transmittance) contain biologically relevant features from deep within the tissue. Lastly, the optical instrument used in this study has been calibrated and validated in this spectral band [27,28].

To estimate the optical properties of the samples, namely $\mu_{a}$ and $\mu_{s}^{\prime }$ , a two-step computational procedure was conducted. In the first step, Monte Carlo simulations from look-up tables of different pairs of ( $\mu_{a}$ , $\mu_{s}^{\prime }$ ) were carried out to produce reflectance and transmittance of articular cartilage samples. In these simulations, the refractive index of the cartilage samples was set to 1.358 [30], and the Henyey-Greenstein phase function was implemented to describe the scattering profile of propagating light. The Henyey-Greenstein scattering phase function estimates the direction of photons after each scattering event as follows:

$$pf_{HG}(\theta ,g)=\frac{1}{4\pi }\frac{1-g^{2}}{{\left(1+g^{2}-2gcos(\theta )\right)}^{\frac{3}{2}}},$$

where θ is the deflection angle and *g* is the scattering anisotropy factor.

Moreover, the geometry and optical properties of the sample holder construct were incorporated into the Monte Carlo simulation. In the second step, an analytical model accounted for the light source strength, integrating sphere throughput and detector efficiency. A detailed description of the two-step computational process is outlined in Foschum et al. [27].

### 2.3 Theoretical Approximation of the Articular Cartilage Absorption Coefficient

In order to be able to compare the estimated values of articular cartilage $\mu_{a}$ with a reference value, a theoretical approximation of articular cartilage $\mu_{a}$ was calculated over the spectral band of 500-1400 nm. To obtain its values, articular cartilage $\mu_{a}$ was considered to be a mixture of its constituents $\mu_{a}$ , weighted by their volume fraction [31] as follows:

$$\mu_{a,\;theoretical}=\;\mu_{a,water}\times V_{water}+\mu_{a,collagen}\times V_{collagen}+\mu_{a,elastin}\times V_{elastin}+\mu_{a,lipid}\times V_{lipid},$$

where $\mu_{a,theoretical}$ is the theoretical approximation of articular cartilage $\mu_{a}$ . $\mu_{a,water}$ , $\mu_{a,collagen}$ , $\mu_{a,elastin}$ , and $\mu_{a,lipid}$ are the absorption coefficients of water, collagen, elastin, and lipid, respectively. $V_{water}$ , $V_{collagen}$ , $V_{elastin}$ , and $V_{lipid}$ are the volume fractions of water (68%), collagen (30%), elastin (1%), and lipid (1%), respectively. The volume fraction values of the components were selected such that they represent the articular cartilage matrix [1]. The absorption coefficients of collagen, elastin, water, and lipid were obtained from the literature [32–36]. An observation removal scheme, based on $\mu_{a,theoretical}$ , was utilized to remove any pair of ( $\mu_{a}$ , $\mu_{s}^{\prime }$ ) from the dataset where the estimated $\mu_{a}$ of articular cartilage sample was deviated significantly from $\mu_{a,theoretical}$ . Lack of the features seen in $\mu_{a,theoretical}$ , signal flattening, and low values of $\mu_{a}$ ( $\leq 10^{-5}$ ) were considered signal distortion and the sample was removed from the dataset. Further information is provided in Supplement 1.

### 2.4 Sensitivity analysis of the articular cartilage optical properties

The sensitivity of the estimated values of optical properties to changes in the surrounding medium properties was investigated by considering the surrounding medium (Fig. 2(C), Material 5) either as water (PBS) or air with the relevant optical properties [28]. As the actual surrounding medium was difficult to control in the experiments, two different types of surrounding medium (air and water) were considered and the overall optical properties were categorized into two classes: 1) with air as the surrounding medium; and 2) with water as the surrounding medium. The objective was to examine how much variation can be observed in the optical properties of articular cartilage if the different surrounding medium was considered. Furthermore, due to the irregular cartilage-bone interface, the shape of the detached articular cartilage differed from that of a perfect cylinder. However, the shape of articular cartilage samples was considered a perfect cylinder in the Monte Carlo simulation (Fig. 2(C)). Using accurate sample volume estimation from microCT imaging. We examined the statistical relationship (statistical correlation) between the volume variation of samples from a perfect cylinder and the variation in the estimated $\mu_{a}$ of articular cartilage compared to $\mu_{a,theoretical}$ . Lastly, we considered different values of scattering anisotropy factor ( g=0.8,0.9,0.99 ) to assess the effect of this parameter on the estimated optical properties. The three values for the parameter *g* are selected based on the possible values that can be considered for a biological tissue like articular cartilage, which favors the forward propagation of light.

Additionally, cartilage optical properties from the literature were collected using curve digitization and discretization with a 1 nm resolution [37]. The extracted optical properties were compared with those estimated in the present study to assess their similarities.

### 2.5 Computational and statistical analysis

All the computational and statistical analyses required for the present report were carried out in MATLAB R2020b and Python v3.7 using standard libraries. The statistical test was: 1) a Student t-test for investigating the normal distribution of the variables and 2) a Pearson/Spearman correlation test (depending on the normal distribution of the statistical variables).

## 3. Results

### 3.1 Sensitivity of the estimated optical properties to the setup parameters

**Fig. 3.**
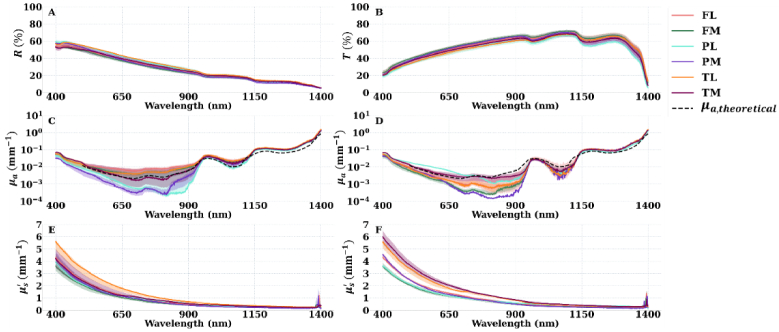
(A) measured reflectance ( R% ) and (B) measured transmittance ( T% ) of articular cartilage. (C) and (D), articular cartilage $\mu_{a}\;(mm^{-1})$ when the surrounding medium is considered air and water, respectively. (E) and (F), articular cartilage $\mu_{s}^{\prime }\;(mm^{-1})$ when the surrounding medium is considered air and water, respectively. *R*, *T*, $\mu_{a}$ , and $\mu_{s}^{\prime }$ of articular cartilage were measured and estimated across anatomical locations: the lateral and medial femur (FL&FM), lateral and medial tibia plateau (TL&TM), and lateral and medial patella (PL & PM) of the bovine knee joint. $\mu_{a,\;theoretical}$ is the theoretical approximation of articular cartilage $\mu_{a}$ . The optical properties were presented in the format of 1^st^ and 3^rd^ quartiles (shaded bands) and the median (solid curve).

**Fig. 4.**
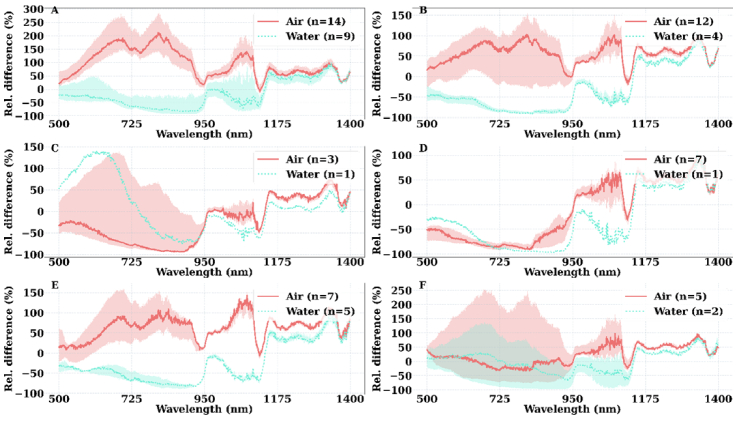
The variation in the relative difference (Rel. difference %) of articular cartilage $\mu_{a}\;(mm^{-1})$ from $\mu_{a,\;theoretical}$ , based on the choice of the surrounding medium (air and water) over different anatomical locations: (A) FL – lateral femur group, (B) FM – medial femur group, (C) PL – lateral patella group, (D) PM – medial patella group, (E) TL – lateral tibia group, and (F) TM – medial tibia group. *n* is the number of samples (observations) per group per surrounding medium. The values were presented in the format of 1^st^ and 3^rd^ quartiles (shaded bands) and the median (solid curve).

**Fig. 5.**
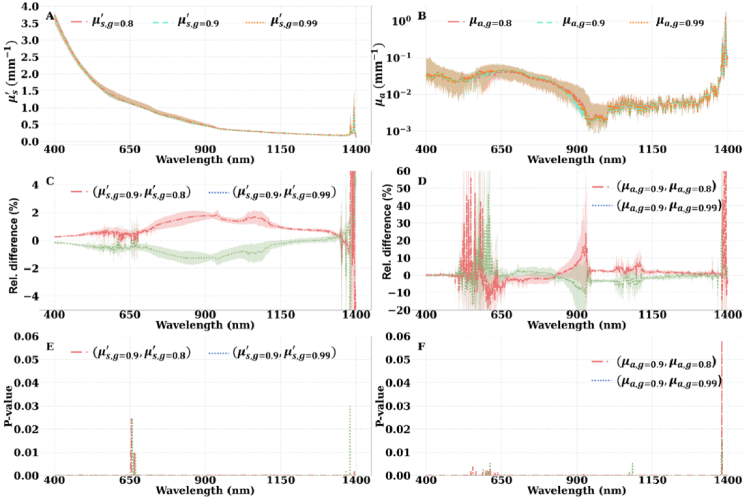
(A) and (B) articular cartilage $\mu_{s}^{\prime }$ and $\mu_{a}\;(mm^{-1})$ from the medial femur (FM), estimated via three *g* values: g=0.8 , g=0.9 , and g=0.99 . (C) the relative difference (Rel. Difference %) between articular cartilage $\mu_{s,g=0.9}^{\prime }$ ( $\mu_{s}^{\prime }$ estimated with g=0.9 ) and $\mu_{s,g=0.8}^{\prime }$ (red curve) and the relative difference $\mu_{s,g=0.9}^{\prime }$ and $\mu_{s,g=0.99}^{\prime }$ (green curve). (D) the relative difference between $\mu_{a,g=0.9}$ and $\mu_{a,g=0.8}$ (red curve) and the relative difference between $\mu_{a,g=0.9}$ and $\mu_{a,g=0.99}$ (green curve). (E) Correlation p-value of $\mu_{s,g=0.9}^{\prime }$ and $\mu_{s,g=0.8}^{\prime }$ (red curve) and correlation p-value of $\mu_{s,g=0.9}^{\prime }$ and $\mu_{s,g=0.99}^{\prime }$ (green curve). (F) Correlation p-value of $\mu_{a,g=0.9}$ and $\mu_{a,g=0.8}$ (red curve) and correlation p-value of $\mu_{a,g=0.9}$ and $\mu_{a,g=0.99}$ (green curve). The values were presented in the format of 1^st^ and 3^rd^ quartiles (shaded bands) and the median (solid curve).

The purpose of this analysis is to determine how much of the variation observed in the estimated optical properties of the samples is due to the natural biological and anatomical variation that exists in the tissues and how much is due to experimental error. Figure 3 illustrates how the choice of the surrounding medium affects the estimated $\mu_{a}$ and $\mu_{s}^{\prime }$ of articular cartilage. As the actual surrounding medium was difficult to control in the experiments, two different types of surrounding medium (air and water) were considered and the overall optical properties were categorized into two classes: 1) with air as the surrounding medium; and 2) with water as the surrounding medium. The measured reflectance, R(%) , and transmittance, T(%) , of the articular cartilage samples are presented in Fig. 3(A) and 3(B). Figures 3(C) and 3(D) depict the articular cartilage $\mu_{a}$ when the surrounding medium is considered either air or water, respectively. Figures 3(E) and 3(F) show the articular cartilage $\mu_{s}^{\prime }$ when the surrounding medium is similarly considered either air or water, respectively. The plotted values of articular cartilage $\mu_{a}$ and $\mu_{s}^{\prime }$ (Fig. 3(C)–3(F)) are obtained based on the Henyey-Greenstein phase function and g=0.9 . Figure 4 depicts the relative difference in articular cartilage $\mu_{a}$ with different surrounding media from $\mu_{a,\;theoretical}$ over different anatomical locations. Table 1 shows the correlation between the sample volume discrepancy and the difference between the estimated $\mu_{a}$ of articular cartilage and $\mu_{a,theoretical}$ . *g* is a wavelength-dependent parameter and is a measure of the scattering angle of the incident beam [31]. Given that *g* of articular cartilage is not known for most of the wavelengths of the 400-1400 nm band, we assumed three values for g=0.8,0.9,0.99 to assess its impact on $\mu_{a}$ and $\mu_{s}^{\prime }$ of articular cartilage. Figures 5(A) and 5(B) illustrate the $\mu_{a}$ and $\mu_{s}^{\prime }$ of articular cartilage estimated based on g=0.8,0.9,0.99 , respectively. Figures 5(C) and 5(D) show the relative difference between ( $\mu_{s,\;\;g=0.8,0.99}^{\prime }$ , $\mu_{a,\;g=0.8,0.99}$ ) from ( $\mu_{s,\;\;g=0.9}^{\prime }$ , $\mu_{a,\;g=0.9}$ ), respectively. Figures 5(E) and 5(F) depict the correlation strength (p-value) between ( $\mu_{s,\;\;g=0.8,0.99}^{\prime }$ , $\mu_{a,\;g=0.8,0.99}$ ) and ( $\mu_{s,\;\;g=0.9}^{\prime }$ and $\mu_{a,\;g=0.9}$ ), respectively.

**Table 1.** Correlation analysis ( φ *& P-value are correlation score and p-value*) between the absolute and relative volume difference of all cartilage samples with the difference of their absorption coefficient and a reference absorption coefficient. Scenarios: A) Air as surrounding medium and microCT-estimated surface diameter, B) air as surrounding medium and drill-bit surface diameter, C) water as surrounding medium and microCT-estimated surface diameter, and d) water as surrounding medium and drill-bit surface diameter**.**

Scenarios	Absolute volume difference		Relative volume difference	
	Correlation parameters		Correlation parameters	
	φ	*P-value*	φ	*P-value*
A	-0.1249	0.419	-0.0424	0.419
B	-0.0845	0.5852	0.0499	0.5852
C	-0.1924	0.4162	-0.1067	0.4162
D	0.3684	0.11	0.4045	0.11

### 3.2 Comparison with the literature

**Fig. 6.**
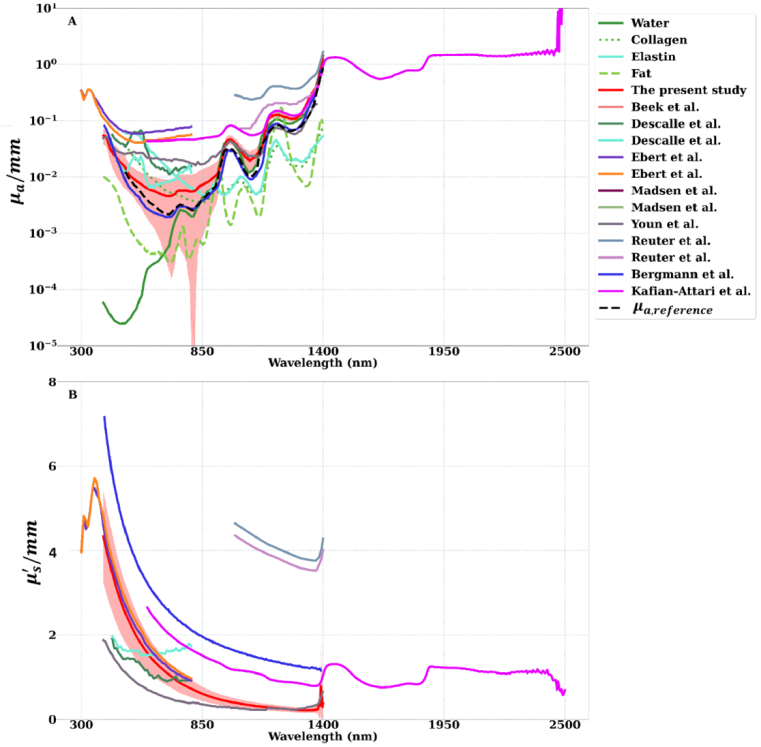
The comparison between the optical properties of cartilage tissues reported in the literature versus the optical properties produced in this study. (A) the reported values of the absorption coefficient ( $\mu_{a}\;[mm^{-1}]$ ). $\mu_{a,theoretical}$ is the reference absorption coefficient (defined in section 2.4). (B) the reported values of the reduced scattering coefficient ( $\mu_{s}^{\prime }\;[mm^{-1}]$ ). The single curves represent the average value of the optical properties. The optical properties of the present study are shown as a band of (average ± standard deviation), with the solid curve depicting the average value over all the anatomical locations with the air as the surrounding medium.

Literature reports of $\mu_{s}^{\prime }$ , $\mu_{a}$ and *g* of various cartilage from different species are presented in Table 2. Figure 6 illustrates the discrepancies and similarities between the optical properties estimated in this study and those reported in the literature. We presented the $\mu_{s}^{\prime }$ and $\mu_{a}$ when air was considered as the surrounding medium. For comparison, $\mu_{a,\;theoretical}$ and $\mu_{a}$ of main articular cartilage constituents, namely water, collagen, lipid, and elastin, were plotted against $\mu_{a}$ of cartilage tissues reported in the literature (Fig. 6(A)).

**Table 2.** Summary of the literature studies on the optical properties of cartilage. $\mu_{a}$ , absorption coefficient; $\mu_{s}^{\prime }$ , reduced scattering coefficient; *g*, scattering anisotropy factor.

Study	Year	Species	Tissue	Optical property	Spectral band	Measurement setup	Computational method
Beek et al. [19]	1997	Rabbit	Articular cartilage	$\mu_{s}^{\prime },g,\mu_{a}$	632.8 nm	Double integrating sphere	Inverse adding-doubling
Ebert et al. [20]	1998	Equine	Articular cartilage	$\mu_{s}^{\prime },\mu_{a}$	300-850 nm	Single integrating sphere	Kubelka-Munk theory
Descalle et al. [21]	1998	Porcine	Articular cartilage, ligament	$\mu_{s}^{\prime },\mu_{a}$	351, 365 nm, 440-800 nm	Fiberoptic setup	Diffuse approximation theory
Madsen et al. [22]	1999	Porcine	Nasal cartilage	$\mu_{s}^{\prime },\mu_{a}$	632.8 nm	Single integrating sphere	Monte Carlo simulation
Youn et al. [23]	2000	Porcine	Nasal cartilage	$\mu_{s}^{\prime },\mu_{a}$	400-1400 nm	Single integrating sphere	Inverse adding-doubling
Reuter et al. [24]	2013	Porcine	Articular cartilage	$\mu_{s}^{\prime },\mu_{a}$	900-1700nm	Single integrating sphere	Kubelka-Munk theory
Kafian-Attari et al. [25]	2020	Bovine	Articular cartilage	$\mu_{s}^{\prime },\mu_{a}$	600-2500 nm	Single integrating sphere	Inverse adding-doubling
Bergmann et al. [26]	2021	Porcine	Ear cartilage	$\mu_{s}^{\prime },\mu_{a}$	400-1400 nm	Single integrating sphere	Monte Carlo simulation

## 4. Discussion

In this study, we aimed to provide accurate values of articular cartilage $\mu_{a}$ and $\mu_{s}^{\prime }$ from different anatomical sites within the knee. As every indirect method of tissue optical properties estimation consists of a measurement step followed by a computational analysis step, there are parameters associated with these steps that could influence the estimation of the optical properties. We investigated the impact of morphological irregularities of the harvested samples, the surrounding medium filled the gap between the samples and the inner walls of the sample holder glass slide (Fig. 2(C) and 2(D)), and *g* of articular cartilage on its $\mu_{s}^{\prime }$ and $\mu_{a}$ .

When water was considered the surrounding medium (Fig. 4), the relative difference of $\mu_{a}$ from $\mu_{a,\;theoretical}$ varied in the range of [-100%, 0%] over the spectral band 500-1135 nm for most of the measurements, whereas the relative difference was mostly between [0%, 100%] over the spectral band 1135-1400 nm where water is the predominant absorber. In contrast, when air was considered the surrounding medium, the relative difference of $\mu_{a}$ from $\mu_{a,\;theoretical}$ predominantly varied in the range of [0%, 250%] over the spectral band 500-1500 nm. Furthermore, for both air and water, the relative difference of $\mu_{a}$ from $\mu_{a,\;theoretical}$ over the spectral band of 1135-1500 nm, became similar in trend and magnitude. This similarity indicates articular cartilage $\mu_{a}$ is invariant to the alteration of surrounding medium properties. One possible explanation for this phenomenon could the strength of $\mu_{a}$ of water ( $\geq 10^{-1}$ ) over the spectral band 1135-1500 nm which leads to higher values of $\mu_{a}$ of articular cartilage, as water is the predominant component of the tissue. In the spectral band of 400-1135 nm, where articular cartilage $\mu_{a}$ is sensitive to variation in the surrounding medium properties, the choice of air as the surrounding medium resulted in larger values for $\mu_{a}$ , compared to its theoretical approximation. On the other hand, when water was considered the surrounding medium, it resulted in smaller values of $\mu_{a}$ in comparison to $\mu_{a,\;theoretical}$ . $\mu_{s}^{\prime }$ of articular cartilage exhibited almost invariance to the choice of the surrounding medium. The difference observed in $\mu_{s}^{\prime }$ under different scenarios in Fig. 3(E) and 3(F) is primarily due to observation removal (Supplement 1).

In the sample preparation stage, due to the curvature of the cartilage-bone interface, separation of articular cartilage tissue from the subchondral bone resulted in an irregular and curved bottom surface for the samples. The extent of this morphological irregularity was different for different anatomical locations. For example, samples from the PL and PM group exhibited the largest morphological irregularity, due to the curvature of the patella, unlike those from the FL group. We postulated that the morphological irregularity would result in heterogeneity of the surrounding medium and possible alteration in articular cartilage optical properties. A possible explanation for different sensitivity of $\mu_{s}^{\prime }$ and $\mu_{a}$ to the surrounding medium could be the significant difference between the mean free path of absorption and scattering events ( ${\mu_{a}}^{-1}$ and ${\mu_{s}}^{-1}$ ). Due to the low values of $\mu_{a}$ of articular cartilage, the mean free path of absorption events becomes large. Thus, for an absorption event to happen, the photons travel longer distances; hence, they may suffer more from the morphological irregularities of the sample. In contrast, for a single scattering event to happen, the photons travel a much lower distance due to the high scattering properties of the tissue. Therefore, they are relatively insensitive to the morphological irregularities of the samples. We postulate that $\mu_{a}$ is influenced by the measurement parameters and the intrinsic biological properties of the samples. As the variation in the experiment parameters becomes more prominent, it is likely to mask the effect of the biological properties of the samples, as shown in Fig. 4(A). In contrast, it is our opinion that $\mu_{s}^{\prime }$ of articular cartilage is strongly influenced by the tissue biological properties.

Given that the spectral properties of the scattering anisotropy factor are not well understood for articular cartilage, we hypothesized that different values of this parameter might induce changes in $\mu_{a}$ and $\mu_{s}^{\prime }$ of articular cartilage. To test this hypothesis, we investigated the relative difference and correlation of articular cartilage $\mu_{a}$ and $\mu_{s}^{\prime }$ when estimated with different *g* values. Our results suggest the relative difference in $\mu_{s}^{\prime }$ was less than 2% for the majority of the wavelengths except for the 1350-1400 nm band, where the variation is due to experimental noise (Fig. 5(C)). Similarly, the median relative difference in $\mu_{a}$ was less than 10% for the majority of the wavelengths except in the 500-650 nm band, at 900 nm, and in the 1350-1400 nm band (Fig. 5(D)). The correlation analysis suggests there is no statistically significant difference between the articular cartilage $\mu_{a}$ and $\mu_{s}^{\prime }$ estimated with different *g* values when g≥0.8 (Fig. 5(E) and 5(F)). This finding can be supported by similar results from Graaf et al. [38] which suggest that for biological tissues, the optical properties are not altered significantly for different values of *g* when g≥0.75 . In addition to *g*, *n* is another wavelength-dependent scattering parameter that could impact the obtained values of articular cartilage $\mu_{a}$ and $\mu_{s}^{\prime }$ . Articular cartilage has refractive index, *n*, in the range of 1.3-1.5, similar to other tissues such as tendon and skin. Bergmann et al. [26] reported the variation of *n* in the range of 1.3-1.5 could result in a relative change of less than 5% for $\mu_{s}^{\prime }$ and less than 13% for $\mu_{a}$ of biological tissues.

The result of statistical analysis (Table 1) between the volume variation of the articular cartilage samples from their perfect cylinder volume and the observed variation in their $\mu_{a}$ from $\mu_{a,theoretical}$ suggests there is no clear statistically significant relationship between these variations. However, we speculate that the variation in the sample volume might not be an ideal candidate for assessing the complexity of morphological irregularity on the estimated optical properties and observed reflectance and transmittance signals. Thus, further analysis, such as Monte Carlo simulations, is needed to implement a realistic 3D geometry of the harvested samples with their inherent shape irregularities to investigate the impact of morphological irregularity on the optical properties and the optical response of biological tissues.

As can be observed in Fig. 6, the $\mu_{a}$ values of articular cartilage reported in the present study are well within the ranges observed in the literature. The early reports of the cartilage $\mu_{a}$ seem to have a higher range of values which become more apparent once compared with $\mu_{a,theoretical}$ . Among the published studies, $\mu_{a}$ of porcine ear cartilage in Bergmann et al.^25^ seems to match exceptionally well with $\mu_{a,theoretical}$ . Although the $\mu_{a}$ values reported in the present study appear to be consistent with the features of $\mu_{a,theoretical}$ , they are slightly larger compared to the $\mu_{a}$ values reported by Bergmann et al. [26] and $\mu_{a,theoretical}$ . Both the present study and that of Bergmann et al.^25^ share the same measurement setup and computational algorithm. They only differed in the sample type, preparation and how the sample preparation matched the measurement setup and computational analysis criteria. The samples extracted in the present study suffer from morphological irregularities due to an irregular interface between the articular cartilage matrix and the underlying subchondral bone. We believe this morphological irregularity contributes to the susceptibility of $\mu_{a}$ of articular cartilage to the surrounding medium properties. In contrast, the porcine ear cartilage samples used in Bergmann et al. exhibited perfect cylinder shape and homogeneity.

When $\mu_{s}^{\prime }$ of articular cartilage is considered, the values reported in the present study deviate significantly from those reported in Bergmann et al. This is most probably mainly due to different collagen fiber orientations in ear cartilage compared to articular cartilage. In contrast, the $\mu_{s}^{\prime }$ values in Ebert et al. [20] were consistent with the present study. The samples used in that study were articular cartilage of the distal femur and the proximal tibia of equine knees. The deviation between the values reported in Bergmann et al. [26] and the present study, and the similarity between values reported in Ebert et al. and the present study, suggest that $\mu_{s}^{\prime }$ of articular cartilage is governed predominantly by collagen fibers (Fig. 5(B)). Given that different cartilage tissues of different species are all predominantly composed of water and a fibrous matrix (with collagen fiber as the main components), they share similar chemical compositions. Hence, the absorption coefficient of different cartilage tissues of different species becomes similar as the tissues share similar chemical composition with analogous quantity. Therefore, it can be observed that $\mu_{a}$ of cartilage is highly dependent on the molecular composition of the tissue and less influenced by the variation in the anatomical site or species.

There are numerous sources of bias and inaccuracy, including incorrect calibration of the optical instrument and the optical measurements, inaccurate model of light propagation such as 1D radiative transfer equation, and setup parameters such as the parameters investigated in the present study. These variables could convolute with tissue optical properties and change their behavior. To the best of our knowledge, inaccurate calibration of the optical instrument and measurements and morphological irregularities of the samples could alter the values of optical properties significantly. Our results suggest that the setup parameters could act as intermediate biases, potentially causing deviations in the optical properties. The impact of some of these setup parameters, such as the scattering anisotropy factor, on the optical properties, are well understood. However, the impact of other parameters, such as the surrounding medium, is poorly understood. Thus, we postulated that the effect of this type of parameter is maximal when the tissue does not comprise strong absorbers or scatterers. For instance, the low absorption coefficient of articular cartilage, which is due to a lack of strong chromophores, is susceptible to these parameters. Using accurate forward models of light propagation enables us to test various states and scenarios for these parameters and to evaluate their impact on the optical response (reflectance and transmittance) and properties (absorption and reduced scattering coefficient) of the tissue.

Additionally, excessive freeze-thaw cycles of biological tissues could alter their structure, as freezing and defrosting of their water content could change the alignment of the fibers of their extracellular matrix [39]. However, this was not investigated in this study. It is worth noting that a strict sample preparation protocol was followed in this study to ensure that all samples were prepared similarly, with the freeze-thaw cycle kept at a minimum before the optical measurements. The other parameter we realized could impact the optical properties is the surface roughness of the bottom end of the samples after detaching the cartilage tissue from the underlying bone. Surface roughness impacts the optical properties by altering the direction of the propagating photons. Since we did not have the means to objectively measure the roughness of the bottom end of the samples, we performed a nominal optical simulation by considering a maximal surface roughness which resulted in a Lambertian distribution of the photons – photons moving in all directions instead of moving only in a forward direction. For a tissue with a nominal thickness of 2 mm, the absorption coefficient showed a maximum of 100% relative change, while the reduced scattering coefficient exhibited a maximum of 12% relative change.

It must be stated that if the goal is to conduct an accurate analysis of articular cartilage tissue composition via its $\mu_{a}$ , the integrating sphere measurement setup has some disadvantages due to the necessities associated with the sample preparation stage. Separating the cartilage component of the osteochondral plug from its subchondral bone results in substantial morphological irregularity, which, in return, leads to a suboptimal estimation of the articular cartilage $\mu_{a}$ . Thus, the reflection-based fiber-optic setups [40] could be an alternative for estimating the $\mu_{a}$ of biological tissues such as articular cartilage, which possesses low absorption efficiency. This is possible because the probed volume can be regulated in the fiberoptic setups (sub-millimeter depth) [40]. Although, these techniques have their limitations including sensitivity to higher moments of scattering phase function and smaller photon path length.

## 5. Conclusion

The present study aims to provide an underpinning theory for how light interacts with healthy articular cartilage. The goal is to provide a basic yet concrete understanding of the optical properties of articular cartilage and its optical response over a wide spectral band such that it could be a reference for further studies on the utilization of methods such as diffuse optical spectroscopy in clinical diagnostics of articular cartilage. Although there are still numerous steps to be taken to reach this goal, we believe the present study provides an understanding of the optical properties and response of healthy articular cartilage. In this report, we provided a reliable estimation of $\mu_{a}$ and $\mu_{s}^{\prime }$ of articular cartilage from different anatomical locations of the bovine knee. In doing so, we performed a sensitivity analysis of the parameters associated with the measurement setup and computational algorithm that could alter the estimated values of articular cartilage optical properties. We investigated the effect of morphological irregularity, the surrounding medium in the sample holder, and the scattering anisotropy factor of articular cartilage. We showed that $\mu_{a}$ is susceptible to variation in the surrounding medium properties. Additionally, our findings suggest that $\mu_{s}^{\prime }$ of articular cartilage is almost invariant to these parameters and thus exhibits robustness to their variation. Moreover, the $\mu_{a}$ and $\mu_{s}^{\prime }$ values reported in the present study are very well within the range reported for the optical properties of cartilage tissues in the literature.

There are numerous sources of bias and inaccuracy, including incorrect calibration of the optical instrument, choice of an inaccurate computational model of light propagation and its parameters, sample morphological irregularity, and sample holder [41–43]. In this study, we emphasized the possible impact of sample morphological irregularity and properties of the sample holder, such as its surrounding medium, as these parameters have not been thoroughly investigated. The mitigation plans for overcoming these challenges, which are dependent on the nature of the samples, sample preparation procedure, measurement setup, and simulation model of light propagation. It is possible that these parameters may not be equally effective for all biological tissues; however, our focus on articular cartilage is motivated by the limited and outdated knowledge of the tissue’s optical properties.

In a future study, we will employ the Monte Carlo simulation method and investigate whether the choice of scattering phase function (Henyey-Greenstein and modified Henyey-Greenstein) could potentially affect the scattering diagram of the photons. We investigate the effect of Rayleigh scatterers and evaluate the penetration depth, depth-origin, and photon path length of the photons to assess the optical response of articular cartilage.

## Data Availability

The data reported in this manuscript is available upon reasonable request.

## Supplemental document

See Supplement 1 for supporting content.
