# Supplementary material for: Impact of experimental setup parameters on the measurement of articular cartilage optical properties in the visible and short near-infrared spectral bands
Source: Biomed Opt Express. 2023 Jun 15;14(7):3397–412. doi: 10.1364/BOE.488801 (PMC10368039; doi:10.1364/BOE.488801)
Supplement: Supplementary file 1 [file boe-14-7-3397-s001.pdf]

Supplemental Document

Biomedical Optics EXPRESS

Impact of experimental setup parameters on the measurement of articular cartilage optical properties in the visible and short near-infrared spectral bands: supplement

IMAN KAFIAN-ATTARI,[1,2,*] ERVIN NIPPOLAINEN,[1] FLORIAN BERGMANN,[3] ARASH MIRHASHEMI,[1] PETRI PAAKKARI,[1,2] FLORIAN FOSCHUM,[3] ALWIN KIENLE,[3] JUHA TÖYRÄS,[1,4,5] AND ISAAC O. AFARA[1,5]

[1] *Department of Technical Physics, University of Eastern Finland, Finland*
[2] *Diagnostic Imaging Center, Kuopio University Hospital, Finland*
[3] *Institute for Laser Technologies in Medicine and Meteorology, University of Ulm, Germany*
[4] *Science Service Center, Kuopio University Hospital, Finland*
[5] *School of Information Technology and Electrical Engineering, University of Queensland, Australia*
[*] *iman.kafianattari@uef.fi*

This supplement published with Optica Publishing Group on 15 June 2023 by The Authors under the terms of the Creative Commons Attribution 4.0 License in the format provided by the authors and unedited. Further distribution of this work must maintain attribution to the author(s) and the published article's title, journal citation, and DOI.

Supplement DOI: https://doi.org/10.6084/m9.figshare.22819925

Parent Article DOI: https://doi.org/10.1364/BOE.488801

Supplementary Material I

Observation removal

Fig. 1 depicts the absorption coefficient of the samples, distorted due to the morphological irregularity of the samples. These observations were removed at the first step of the analysis as including them would cause deviation in the absorption coefficient of articular cartilage. Furthermore, a theoretical absorption coefficient of articular cartilage with typical values of volume fraction for its components was estimated as follows:

$$\mu_{a,\,theoretical} = \mu_{a,water} \times V_{water} + \mu_{a,collagen} \times V_{collagen} + \mu_{a,elastin} \times V_{elastin} + \mu_{a,lipid} \times V_{lipid},$$

where $\mu_{a,theoretical}$ is the theoretical approximation of articular cartilage μ_a. $\mu_{a,water}$, $\mu_{a,collagen}$, $\mu_{a,elastin}$, and $\mu_{a,lipid}$ are the absorption coefficients of water, collagen, elastin and lipid, respectively. V_{water}, $V_{collagen}$, $V_{elastin}$, and V_{lipid} are the volume fractions of water (68%), collagen (30%), elastin (1%), and lipid (1%), respectively. $\mu_{a,\,theoretical}$ is shown in Fig. 1 to emphasize distortion of μ_a of removed samples. Lack of the features seen in $\mu_{a,theoretical}$, signal flattening, and low values of μ_a ($\leq 10^{-5}$) were considered signal distoration.

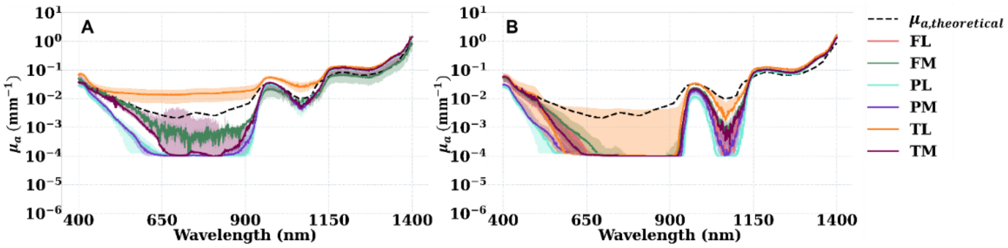

Figure 1. The signal distortion in the estimated absorption coefficient (μ_a [mm^{-1}]) of the removed articular cartilage samples. The signal saturation is mostly due to morphological irregularity of the samples occurred in the sample preparation stage. Articular cartilage μ_a: (A) air as surrounding medium; (B) the water as surrounding medium. $\mu_{a,\mathrm{theoretical}}$ is the theoretical approximation of articular cartilage absorption coefficient. The anatomical locations are the lateral and medial femur (FL, FM), lateral and medial patella (PL, PM), and lateral and medial tibia (TL, TM) of the bovine knee. The dataset is presented as first and third quartiles (shaded area) and median (the solid curve).
